# Synthesis of Multiwall Boron Nitride (BN) Nanotubes by a PVD Method Based on Vapor–Liquid–Solid Growth

**DOI:** 10.3390/ma13040915

**Published:** 2020-02-19

**Authors:** Hao Guo, Yonggang Xu, Hetuo Chen, Zhengjuan Wang, Xiaojian Mao, Guohong Zhou, Jian Zhang, Shiwei Wang

**Affiliations:** 1 State Key Laboratory of High Performance Ceramics and Superfine Microstructure, Shanghai Institute of Ceramics, Chinese Academy of Sciences, Shanghai 200050, China; guohao@student.sic.ac.cn (H.G.); wzhj926@mail.sic.ac.cn (Z.W.); sic_zhough@mail.sic.ac.cn (G.Z.); jianzhang@mail.sic.ac.cn (J.Z.); 2 Center of Materials Science and Optoelectronics Engineering, University of Chinese Academy of Sciences, Beijing 100049, China; 3 CAS Key Laboratory of Transparent and Opto-Functional Inorganic Materials, Shanghai Institute of Ceramics, Chinese Academy of Sciences, Shanghai 201899, China

**Keywords:** Boron nitride nanotubes, vapor–liquid–solid growth, physical vapor deposition

## Abstract

Multiwall boron nitride (BN) nanotubes were synthesized by a novel physical vapor deposition (PVD) method, in which the BN nanotubes grow on a compact substrate composed of AlN, γ-Al_2_O_3_, Y_2_O_3_, and carbon powders. The obtained BN nanotubes assemble in an orderly manner with a typical length of over one millimeter and a diameter of one-hundred nanometers. The hollow multiwall tubes have a spherical tip, which is presumed to be a liquid drop at the synthesis temperature, indicating the vapor–liquid–solid (VLS) growth mechanism.

## 1. Introduction

Boron nitride (BN) nanotubes are structurally analogue to carbon nanotubes—they substitute B and N atoms entirely for C atoms in graphitic-like sheets with nearly no change in the atomic spacing [1,2,3]—but exhibit superior thermal and chemical performances to carbon-based materials [4]. Hence, there has been a growing interest in utilizing BN nanotube materials in specific applications in high-temperature or chemically hazardous environments in recent years [3]. In addition, BN tubes and continuous fibers can also be applied to reinforce electromagnetic windows because of their low dielectric constant [5].

Rubio et al. first proposed the idea of rolling up individual hexagonal boron nitride (h-BN) sheets to form BN nanotubes in 1994 [1,2]. Zettl et al. successfully obtained BN nanotubes by an arc-discharged method that used refractory metal as electrodes in 1995, inspired by the successful preparation of carbon nanotubes [6]. However, the yield of the resultant nanotube material was limited. Afterwards, many other synthesizing methods were developed, such as laser ablation [7], chemical vapor deposition [8], the heating of milled mixtures [9,10], the plasma-jet method [11], and atom deposition [12]. However, most of these methods are too complicated to be suitable for industrial manufacturing.

In 1999, Kukovitsky et al. developed a vapor–liquid–solid (VLS) method to grow carbon nanotubes on a supported catalyst, in which the arc discharge between graphite electrodes was used as the carbon vapor source and nickel particles were used as a catalyst [13]. However, the morphology of the resulting carbon products was variable because of the unstable vapor source. A similar VLS growth method was also applied in the synthesis of BN nanotubes. In 2011, Li et al. [14] synthesized BN nanotubes on stainless-steel substrates by reacting Boron and Fe_2_O_3_ at 1300 °C under a flowing ammonia atmosphere. The B–N–O–Si–Mn particles were considered to be the catalyst for the growth of BN nanotubes. Afterwards, Wang et al. [15] developed an optimized self-cracking catalyst VLS growth mechanism to manufacture BN nanotubes by using boron-containing precursors. The BN nanotubes were formed on the surface of glass fabrics, which were used as a product collecting framework and growth catalyst. The critical issue of this method was to synthesize the boron-containing precursor in advance via the dilution self-propagation high-temperature synthesis and post- annealing (SHS) method. It is known that h-BN will evaporate at an elevated temperature [16]. In this case, physical vapor deposition might be a possible method to manufacture BN nanotubes. The critical issue is to develop a liquid catalyst to control the deposition of BN vapor.

In the present work, a physical vapor deposition method based on the VLS-growth mechanism was developed for the growth of the BN nanotubes by using a Y–Al–O ternary compound as the active catalyst. The structure of the BN tubes was analyzed by scanning electron microscopy (SEM), transmission electron microscopy (TEM), an X-ray diffractometer (XRD), and energy dispersive spectroscopy (EDS). The growth mechanism of the BN tubes was also explored.

## 2. Methods and Materials

A h-BN ceramic disk, which contained 0.5 wt.% Al_2_O_3_, 0.2 wt.% CaO, and 1.0 wt.% ZrO_2_, was used as the resource of B and N. High purity AlN, γ-Al_2_O_3_, Y_2_O_3,_ and carbon were mixed by ball milling in ethanol with the weight ratio of 100:100:3:80. After drying, the mixture was compressed into a pellet with a diameter of 40 mm by cold isostatic pressing at 200 MPa for 5 min. The above compact pellet was used as the substrate for the growth of BN tubes. The substrate was placed on the h-BN ceramic disk directly, which was then put inside a covered alumina crucible. A vertical slot of about 5 mm in width was designed on the alumina crucible wall to produce a system with a semi-open atmosphere. The experiment was performed in a graphite furnace. The furnace was heated with a heating rate of 10 °C/min to 1850 °C and held for 4 h in a flowing N_2_ atmosphere.

The morphology of the nanotubes was characterized by scanning electron microscopy (SEM, CrossBeam 550, ZEISS Co., Jena, Germany). The phase structure was analyzed by an X-ray diffractometer (XRD, D8-Advance A25, Bruker Co., Karlsruhe, Germany) with Cu K_α_ radiation in the 2θ range from 10° to 80° with the step size of 0.02°. Transmission electron microscopy (TEM, JEM-2100F, JEOL Ltd., Tokyo, Japan) was used for microstructure analysis. An energy spectrum analysis was carried out by energy dispersive spectroscopy (EDS, Oxford X-Max 80, Oxford Instruments Co., Oxford, UK) attached to the SEM and TEM instruments.

## 3. Results and Discussion

**Figure 1:**
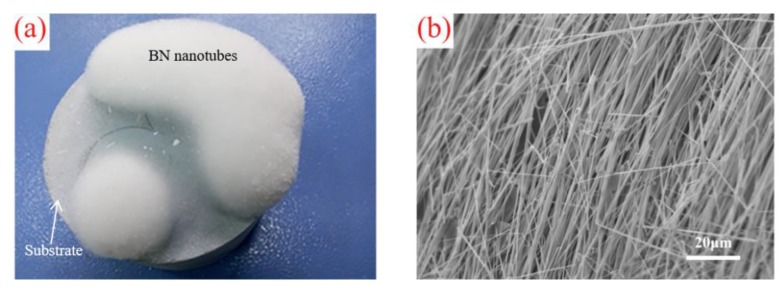
Photograph (**a**) and SEM photograph (**b**) of the obtained boron nitride (BN) nanotubes.

The obtained white flocculent BN nanotubes attached to the surface of the substrate are demonstrated in Figure 1a. It is notable that the length of the nanotubes is over one millimeter and the volume of the products indicates that the BN nanotubes could be manufactured on an industrial scale.

The SEM microstructure of the resulting BN nanotubes is shown in Figure 1b. It is obvious that the nanotubes assembled in an orderly manner with a smooth surface. The morphology of the present nanotubes, both for a single tube’s morphology and their assembly, is much better than the products manufactured by earlier synthesis methods [17,18].

**Figure 2:**
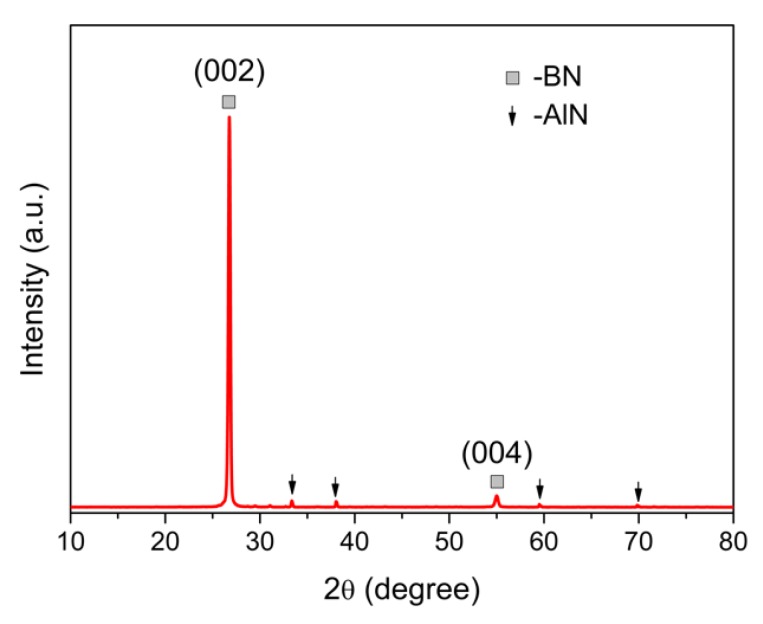
XRD patterns of the obtained BN nanotubes.

Figure 2 shows the XRD pattern of the flocculent BN nanotube materials after being pressed flatly. It is clear that a strong diffraction peak of the (002) plane for h-BN was detected, combined with a petty drum of the (004) plane. Other weak diffraction peaks were assigned to AlN crystals, which signals AlN contamination in the obtained BN nanotubes. Compared with the standard card (pdf-45-0893), many other diffraction peaks disappeared. Because the nanotubes were pressed to lie down for XRD measurement, the surface of the nanotubes is the (001) plane, which indicates that the nanotubes could be regarded as rolled-up hexagonal h-BN sheets.

**Figure 3:**
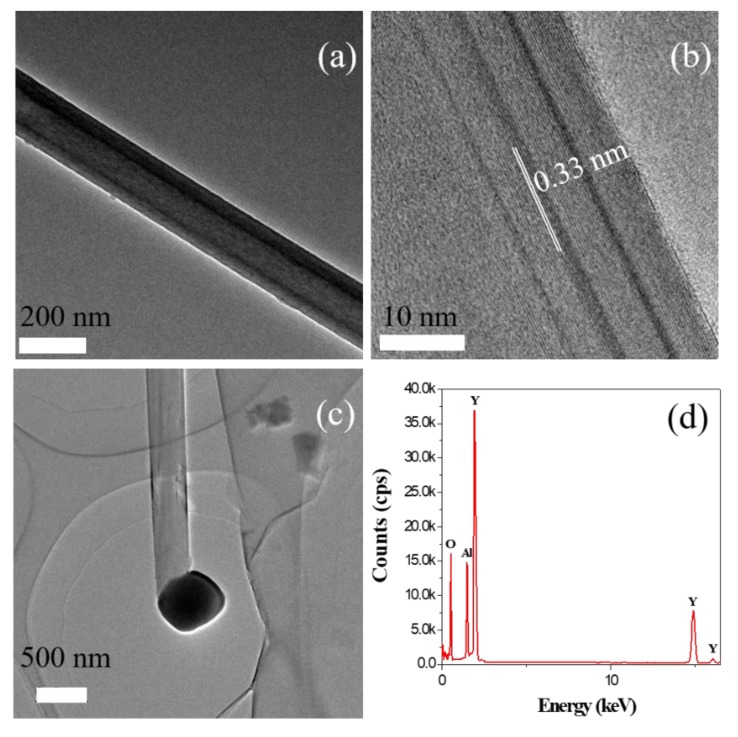
TEM microstructures of (**a**) the BN nanotube, (**b**) the tube wall, (**c**) nanotubes of the end tip, and (**d**) the EDS pattern of the spherical end tip.

The microstructure of the BN nanotubes was confirmed by TEM, as shown in Figure 3. It is obvious that the hollow BN nanotube exhibits a smooth surface with a diameter of about 175 nm, as shown in Figure 3a. Figure 3b shows the multiwall structure with an interlayer distance of about 0.33 nm, which is close to the (002) plane of h-BN crystals [19]. This microstructure is coincident with the results of the XRD patterns shown in Figure 2. The TEM microstructure of the top end of the resulting BN nanotubes is shown in Figure 3c. A dark spherical tip was found clearly on top of the nanotubes. It was composed of Y, Al, and O elements as indicated by EDS, as shown in Figure 3d. This is a typical characteristic of one-dimensional (1-D) materials growth via the vapor–liquid–solid growth method, where the tip acts as the catalyst [20].

**Figure 4:**
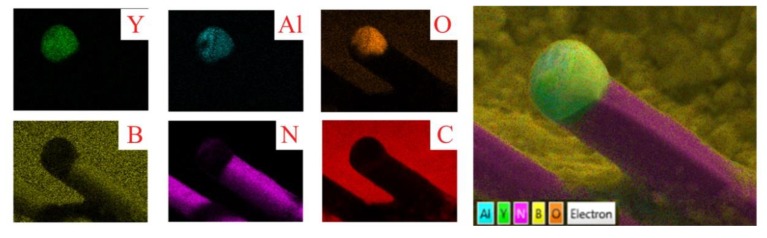
Element scanning maps of a typical BN nanotube under SEM: single element distribution (left), and combination maps (right).

Figure 4 shows the element scanning maps of the BN nanotube close to the tip by EDS in SEM. It is clear that the nanotube is BN and the spherical tip has the components of Y_2_O_3_ and Al_2_O_3_. This indicates a typical mechanism of VLS growth, which is normally used for the growth of nanowires and nanowhiskers [21]. In this process, the vapored species condenses preferentially into the catalytic liquid drops and deposits serially from the supersaturated liquid drops.

In the present synthesis system, the substrate contains Y_2_O_3_ and Al_2_O_3_, which have a eutectic point at 1780 °C [22,23]. Hence, when heated up to 1850 °C, some eutectic liquid is formed in the substrate. It was noted that the substrate also contains carbon, which will react with Al_2_O_3_ and N_2_ gas at elevated temperatures, producing CO gas. The designed semi-open system keeps a weak oxide atmosphere inside the alumina crucible, which allows for the survival of the residual Al_2_O_3_. From the Y_2_O_3_–Al_2_O_3_–AlN pseudo-ternary phase diagram [24], it can be found that AlN may also dissolve into the eutectic liquid. Because the amount of Y_2_O_3_ is very low, there should be very small liquid drops formed in the substrate at the synthetization temperature.

**Figure 5:**
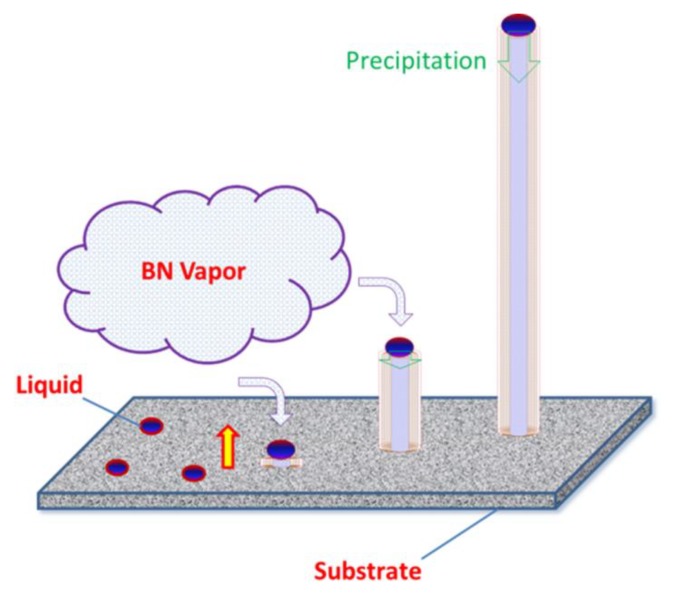
A schematic diagram of vapor–liquid–solid (VLS) growth for BN nanotubes.

The schematic diagram of the BN nanotubes synthesized by thermal evaporation is illustrated in Figure 5, which demonstrates the VLS mechanism of BN nanotubes in this research. As shown in Figure 5, firstly, the eutectic liquid drops of Y_2_O_3_–Al_2_O_3_, which may have contained AlN, formed on the surface of the substrate at the synthesis temperature of 1850 °C. Then, the vapored BN species dissolved into the liquid drops and condensed in preferential sites. Unlike the normal VLS growth process, the precipitation of BN atoms was along the (001) plane rather than stacking layer by layer. The extension of BN sheets was restricted to inside the liquid drops on the scale of 100 nm. Hence, the BN sheets rolled up to become the embryo of the BN nanotube. Serially, the supersaturated BN atoms in liquid drops precipitated along the nanotubes, which resulted in the growth of BN nanotubes. The sequential steps of evaporation, dissolution, and precipitation extended the BN nanotubes to one millimeter in length. The liquid drop diameter determined the diameter of the BN nanotubes, where the BN sheets rolled up at the beginning. Modifying the liquid drops to control the morphology of the final BN nanotubes may be valuable in the future.

## 4. Conclusions

In summary, multiwall BN nanotubes were synthesized by a PVD method, where h-BN ceramics were used as a BN source. The resulting BN nanotubes exhibited a smooth surface and were arranged in an orderly manner. The spherical Y_2_O_3_–Al_2_O_3_ eutectic tip on the top of the BN nanotubes indicated the VLS growth mechanism. Given the synthesis conditions, the Y–Al–O ternary liquid drops were formed on the surface of the substrate, and then acted as a catalyst for the growth of BN nanotubes. It is believed that this simple fabrication method can effectively accelerate the industrial production of BN nanotubes in the near future, since the current fabrication processes are mainly based on complex chemical methods.
